# Short-term cold-water immersion does not alter neuromuscular fatigue development during high-intensity intermittent exercise

**DOI:** 10.3389/fphys.2022.1061866

**Published:** 2023-01-24

**Authors:** Robin Faricier, Olivier Haeberlé, Marcel Lemire

**Affiliations:** ^1^ School of Kinesiology, The University of Western Ontario, London, ON, Canada; ^2^ IRIMAS UR UHA 7499, University of Haute-Alsace, Mulhouse, France; ^3^ Faculty of Sport Sciences, University of Strasbourg, Strasbourg, France; ^4^ Faculty of Medicine, Translational Medicine Federation (FMTS), University of Strasbourg, Strasbourg, France

**Keywords:** neuromuscular, fatigue, cold water-immersion, pre-cooling, exercise

## Abstract

Aims: Pre-exercise cold-water immersion affects physical performance under ambient environment, however the mechanisms leading to this decrease remains to be elucidated. The purpose was to determine whether short-term lower-body immersion in cold water could induce acute changes in the development of neuromuscular fatigue after high-intensity exercise. Methods: Ten participants performed on two separate visits a fatigue task (60 intermittent isometric maximal voluntary contractions maintained over 3 s and spaced by 2 s of recovery) once after lower-body cold-water immersion (Pre-Cooling, 6 min at 8.9°C ± 1.6°C) and another time without prior immersion (Control). Before and after the fatigue task, neuromuscular function was assessed during voluntary or evoked contractions (electrical stimulation performed on the femoral nerve) on contracted and relaxed on knee extensor muscles. Results: No differences in neuromuscular fatigue were measured between Pre-Cooling and Control conditions, despite maximal voluntary contraction reductions (−49 and −48%, respectively, both *p* < 0.05), peripheral contractile capacities (both -28%, *p* < 0.05). Additionally, rate of perceived exhaustion increases over time for both conditions (both *p* < 0.05) with differences in the time course. Discussion: Lower body immersion in extreme cold water for a short period of time was not a sufficient stimulus to induce a significant disruption of human body homeostasis: neuromuscular function was not significantly altered during a maximum intensity fatigue task.

## Introduction

The influence of cold exposure on neuromuscular function has attracted much interest in order to improve human performance (Racinais and Oksa, 2010). During cold water exposure, the human body regulates its own temperature with the activation of thermoregulatory systems allowing the maintenance of its central temperature and preservation of cellular homeostasis, such as skin blood vessels vasoconstriction to reduce the loss of heat, and increase in metabolic rate (shivering) to increase heat production (Castellani and Young, 2016).

While exposure to cold prior to long-duration endurance exercise, also called pre-cooling, appears to be an effective method to improve performance, especially in hot environment (Maia-Lima et al., 2017), induced hypothermia (i.e., decrease in body temperature) after cold exposure reduces performance capacity during short and explosive exercise (Fischer et al., 2009; Racinais and Oksa, 2010; Douzi et al., 2019).

The acute response after cold water immersion of a body part (e.g., clavicle, hand and forearm) or whole-body is a decrease in the force-producing capacity of the studied skeletal muscle groups (Bigland-Ritchie et al., 1992; de Ruiter et al., 1999; Cahill et al., 2011; Solianik et al., 2015). Lowering muscle temperature *via* a passive cooling procedure, using whole-body immersion in 14°C water for ∼140 min, reduced by about 30–40% skeletal muscle force-generating capacity in female and male individuals (Solianik et al., 2015). Indeed, a strong dose-effect relationship is present between skeletal muscle performance and temperature (Racinais and Oksa, 2010), and when the muscle temperature decreases from 36°C to 22°C, the degree of skeletal muscle function impairment decreases rather linearly (de Ruiter et al., 1999).

Changes in skeletal muscle contractile properties under hypothermia conditions have been associated with altered metabolic processes such as reduced excitation-contraction coupling efficiency (i.e., calcium-release related) and impaired enzymatic activity (Davies and Young, 1983; Bigland-Ritchie et al., 1992; de Ruiter et al., 1999). Reduced skeletal muscle performance can also arise from an impairment in the central command function, such as a neural drive dysfunction (Franssen and Wieneke, 1994). The reduction in motoneuron conductivity velocity in response to cold stress (Franssen and Wieneke, 1994) lowers the total motor unit recruitment response to a given action-potential train and *per se* skeletal muscle force-generating capacity.

However, a pre-cooling strategy appears to have a beneficial effect on the rate of central fatigue development during high-intensity exercise (de Ruiter et al., 1999; Cahill et al., 2011; Brazaitis et al., 2012). While, under skeletal muscle hypothermia condition, the metabolic turnover speed becomes slower, the frequency of the motoneuronal discharge is preserved (Bigland-Ritchie et al., 1992). It has been suggested that the reduction in metabolite accumulation within skeletal muscle cells following cold exposure might lower inhibitory feedback signals from metabosensitive afference to the central command (Cahill et al., 2011).

Most studies investigating the effect of cold-water immersion on neuromuscular function immersed their participants for a long period of time (i.e., >30 min) (Cahill et al., 2011; Brazaitis et al., 2012; Solianik et al., 2015), while time is a crucial component of preparation efficiency. Therefore, it seems interesting to investigate whether similar neuromuscular function responses might be observed when immersed in colder water for a shorter time. Thus, the aim of the present study was to determine whether a short-term lower-body immersion in cold water could induce acute changes in the development of neuromuscular fatigue after high-intensity exercise. It was hypothesized that a more pronounced decrease in force production capacity (i.e., lower absolute torque values) but a lower rate of fatigue development (i.e., difference between pre and post values) would be observed after short-term lower body immersion in cold water.

## Methods

### Participants

Ten voluntary participants took part in the study (height: 1.76 ± 0.08 cm; weight: 73.4 ± 9.1 kg; body mass index: 23.6 ± 2.6 [mean ± SD]). All participants were physically active and injury-free for at least 6 months prior to the beginning of the study. Individuals aged 18–55 years and regularly exercising were eligible for inclusion in the study. However, individuals on medication, smokers, or with evidence of cardio-respiratory, metabolic or lower-limb musculoskeletal pathologies were not included. The research protocol was approved by the local ethics committee (CER-2021-42). Clear written explanations of the benefits and risks incurred of the experiment were presented to the participants. Then, the participants returned the consent form signed, and could withdraw from the project at any time.

### Experimental design

Neuromuscular function before and after a fatigue task was assessed. This fatigue task consisted of 60 intermittent isometric maximal voluntary contractions (iMVC) maintained over 3 s and spaced by 2 s of recovery. During the experimental condition (Pre-Cooling), participants were immersed in a bath (form4; cryocontrol, Castanet Tolosan, France) for 6 min containing cold water (8.9°C ± 1.6°C) just before performing the fatiguing task. During the control condition, participants rested for 6 min instead of the 6 min cold water immersion. The immersion duration was determined from our pilot trials considering bath temperature and participant tolerability. One week separated both conditions, and their order was randomly assigned. To suppress the circadian effect on performance, experiments were performed at the same time of day and under the same laboratory environmental condition (temperature: 20.2°C ± 0.6°C and humidity: 57.0 ± 2.4%). In addition, participants were advised to restrict themselves to unusual activities and to take the same meal for at least 24 and 3 h (respectively) prior coming to the laboratory.

### Neuromuscular testing protocol

Assessment of neuromuscular function was executed on the dominant knee extensor muscles (i.e., right lower limb for all participants) using a commercially available dynamometer (Con-Trex MJ; CMV AG, Dübendorf, Switzerland). In the pre-neuromuscular function assessment, the first step was to determine the intensity of the electrical peripheral nerve stimulation on the femoral nerve (DS7A, Digitimer, Hertfordshire, United-Kingdom). While the quadriceps muscle was still relaxed, percutaneous electrical stimulations were delivered to evoke muscle contraction. The intensity of the electrical stimulations was progressively increased until reaching a plateau in the evoked contraction force (maximal twitch response–Pt). Each electrical stimulation was separated by 30 s of rest. Then, participants performed a warm-up composed of progressive submaximal voluntary contractions (from 50 to 70% of perceived iMVC), prior to execute 3 iMVC (sustained for 4 s) separated by 1 min of resting period. A 100 Hz doublet electrical nerve stimulation was superimposed over the force plateau of the last 2 iMVC, followed by three distinct electrical nerve stimulations (a 100 Hz doublet, a 10 Hz doublet, and a single twitch) delivered on the relaxed muscles. The peak force value of the first iMVC was used as a reference to ensure participants produced maximal contraction during the following iMVCs with electrical stimulations. At the end of the fatiguing, the same electrical procedure was applied over the last iMVC of the fatiguing exercise (60^th^ contraction) to assess the status of post-exercise neuromuscular function. Moreover, frontal temperature (FLUKE, 566; Dubai, United Arab Emirates, precision of 0.1°C) and the rate of perceived exhaustion (RPE) were measured every 10 contractions with a scale from 6 to 20 (Borg, 1970). Frontal temperature was consistently measured about 20 cm from the participants’ front within the supraorbital incisure. During the pre-cooling condition, participants were immediately (i.e., <1 min) towel dried and settled on the dynamometer.

### Force recordings

Voluntary and evocated force were directly measured by the dynamometer. The same chair settings were used at each visit for a given participant. During each contraction, participants were seated on the chair with both hip and knee angles bent at 90°. Non-compliant straps were used to fix the participant’s position during contractions at the trunk, hip, and quadriceps levels. Participants had real-time visual access to the force, voluntary or evocated, produced information, and received strong verbal encouragement to push as hard as possible. Electrical nerve stimulations were applied using a cathode electrode (3 cm × 3 cm, Ag-AgCl, Mini-KR, Contrôle-Graphique, Brie-Comte-Robert, France), placed on the femoral triangle, at the stimulation site which resulted in the maximal force output. The anode electrode (70 mm × 50 mm) was placed on the gluteal fold. To ensure consistency between sessions, the settings of each participant were exactly reproduced.

### Data analysis

The highest peak force value was used to determine the iMVC of the knee extensors. To assess the evolution of the central neuromuscular component of the contraction properties, the voluntary activation (VA) was computed using the amplitudes of the superimposed and resting twitches at 100 Hz (with VA in percentage and twitches amplitude in N·m):

$$VA=\left(1-\frac{100\;Hz\;Superimposed\;Twitch\;Amplitude}{100\;Hz\;Resting\;Twitch\;Amplitude}\right)x100.$$ (1)

Additionally, several peripheral parameters were measured to evaluate the effect of the fatiguing task on the muscular contractile properties: Db 100, Pt, and Db100:10 ratio. The latter ratio was computed to investigate the presence of low-frequency fatigue. All the neuromuscular measurements were recorded using Labchart 8 pro (ADInstruments, Bella-Vista, Australia).

### Statistical analysis

After normality and sphericity assumptions verification, two-factor repeated measurement ANOVAs were performed to investigate the time and condition main effects and their interaction effect (time*condition) for each selected parameters (iMVC, VA, Db100, Db 100:10 ratio, Pt, RPE, and frontal temperature) (Jamovi 1.6.23, Sydney; Australia). A *p*-value lower than 5% was considered statistically significant.

## Results

The neuromuscular responses before and after fatiguing task are displayed in Table 1. No main condition or time*condition interaction effect appeared for any of the assessed neuromuscular parameters (all *p* > 0.05). However, except for VA (*p* = 0.077), a decrease from before to after the fatiguing task occurred for the iMVC, Db100, and Pt, while Db 100:10 ratio increased (all *p* < 0.05 for the main time effect).

**TABLE 1:** Neuromuscular response before and after the fatiguing task (60 intermittent iMVC maintained over 3 s and spaced by 2 s of recovery).

	Pre-cooling		Control	
	Before	After	Before	After
iMVC (N·m)	200 ± 39	101 ± 24	201 ± 45	102 ± 24
VA (%)	86.3 ± 7.5	77.0 ± 17.6	82.9 ± 9.6	69.7 ± 21.2
Db 100 (N·m)	68.1 ± 22.0	49.1 ± 20.7	75.2 ± 29.1	59.0 ± 23.4
Pt (N·m)	44.2 ± 12.8	28.7 ± 8.1	46.6 ± 11.0	32.8 ± 10.1
Db 100:10 ratio	0.95 ± 0.11	1.15 ± 0.20	1.01 ± 0.09	1.25 ± 0.19

Data are presented as Mean ± SD; iMVC: isometric maximal voluntary contraction; VA: voluntary activation; Db 100: doublet 100 Hz; Pt: peak twitch; Db 100:10 ratio: doublet 100 over 10 Hz ratio.

**FIGURE 1:**
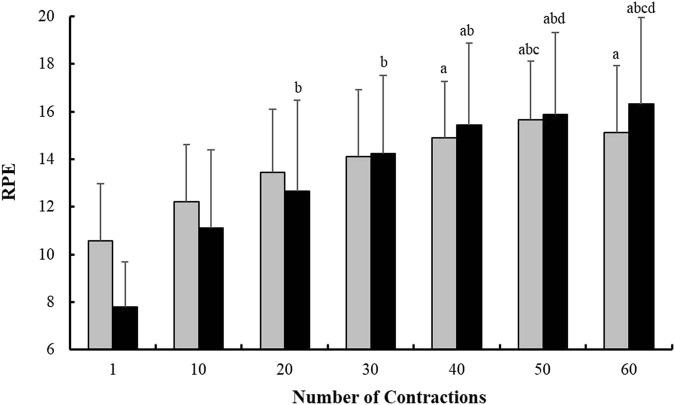
Evolution of the Rate of Perceived Exhaustion (RPE) response measured every 10 isometric Maximal Voluntary Contraction (iMVC) during the fatiguing task for each condition (Pre-cooling: white bars, and Control: black bars). a *p <* 0.05 *vs*. iMVC 1; b *p <* 0.05 *vs*. iMVC 10; c *p <* 0.05 *vs*. iMVC 20; and d *p <* 0.05 *vs*. iMVC 30.

Additionally, as for the neuromuscular measurements, no main condition effect was found in RPE values (*p* > 0.05), but a main time and time*condition interaction effects appeared (*p* < 0.001 and *p* = 0.030, respectively) (Figure 1).

Finally, the frontal temperature measured at the end of both the bath and the fatiguing tasks was not affected by neither the condition nor the time (from 36.7°C to 36.5°C, and from 36.6°C to 36.3°C, for the pre-cooling and the control condition, both *p* > 0.05).

## Discussion

This study was the first to investigate the effect of short-term lower-body immersion in cold water on neuromuscular fatigue development following high intensity intermittent exercise. According to the results of the present study, 6-min of lower-body immersion in ∼8.9°C water does not alter fatigue development. No differences in the acute changes were observed following the repetition of 60 iMVC, preceded or not by a cold-water immersion. This result provides new insight into understanding how a short term lower-body exposition to cold temperature affects human performance during a dynamic single-leg exercise and the associated adjustment in neuromuscular function.

In the present study, the decrease in iMVC, by approximately a half in both conditions (−49 *vs.* −48% for pre-cooling and control condition, respectively), is comparable with those observed in the literature under ambient condition and isolated muscle group (McKenzie et al., 1992; Burnley, 2009). However, the lack of difference between conditions contradicts the results of studies involving local (Cahill et al., 2011; Brazaitis et al., 2012) or whole-body (Solianik et al., 2015) cold water immersion. The duration of the cold exposition is probably the main determinant in the discrepancy between the previous and the present study. While a 10-min cold exposition in an ice bag is enough to impair human motor performance, the immersion in a same ice bag for 3 min does not produce any alteration (Fischer et al., 2009). As short as a 6-min stimulus was probably not sufficient to generate profound physiologic disruption to homeostasis and decrease body temperature that could alter force-generating capacity, such as a vasoconstriction and an increase in a metabolic rate. However, despite the close positive relationship between skin and muscle temperature (de Ruiter et al., 1999), without direct measurement of muscle tissue temperature it is challenging to make a direct comparison with previous studies. Indeed, the temperature response to cold water exposition differs depending on the site of measurement: for a given stimulus, the degree of temperature reduction is greater at skin level compared to muscle and rectal level (Solianik et al., 2015). Indeed, although in some participants frontal temperature was reduced following the pre-cooling protocol, the level of diminution might not have been sufficient to lower muscle temperature.

Additionally, this study highlights in a novel way the involvement of central and peripheral fatigue mechanisms during precooling. After lower-limb cold-water exposure, the loss in the general ability to generate force was accompanied by a reduction in muscular contractile capacity. Nevertheless, the voluntary activation capacity remains unchanged, without difference between conditions. The intensity-dependent contribution of peripheral and central fatigue is now largely demonstrated (Thomas et al., 2016). High intensity exercises required energy production from substrate-levels phosphorylation to cover the energy demand. However, by-products, such as hydrogen ions and phosphate inorganic, associated are detrimental for the proper functioning of active skeletal muscles (Black et al., 2017). The preservation of central command integrity might be explained by the close inter-regulatory relationship between peripheral and central compounds following the pre-cooling condition. The reduction in neuronal drive conductivity to muscle tissue (Franssen and Wieneke, 1994), in addition to the diminished speed of the skeletal muscle contractile process (de Ruiter et al., 1999), might have both contributed to reduced metabolic activity in response to cold stress. A slower muscle energetic rate would limit metabolite accumulation and the firing rate of inhibitory feedback to the central system from metabosensitive afferents. Nevertheless, more investigations are warranted on that topic to extend our understanding regarding the degree of central and peripheral compounds’ contribution to the impairment in skeletal muscle force-generating performance under hypothermia conditions. From a practical point of view, adequate physiological adaptations to maintain proper thermal balance are essential to protect vital body systems and functions. Deviation from the body temperature operating point impairs physical performance, especially during activities in hot environments where exercise-induced hyperthermia is exacerbated and precipitates exhaustion (González-Alonso and Calbet, 2003). The development of new practical methods, such as pre-cooling, is consequently warranted to reduce heat strain and improve exercise tolerance (Ruddock et al., 2017). The more rapid pre-cooling strategy used in this study might represent an efficient protocol to limit the physical exertion in increasing body temperature during high-intensity exercise and/or in a hot environment by maintaining neuromuscular function performance.

## Conclusion

In conclusion, the present study does not show any differences in terms of the neuromuscular function alteration following a short term lower-body cold water immersion. Perhaps a combination of short term and extreme cold-water exposure was not a sufficient stimulus to induce a significant disturbance in the human body’s homeostasis, and consequently on the neuromuscular function. Nevertheless, the result of the study might present preliminary data on the contribution of time exposure and cold temperature on fatigue development. Indeed, a drastic decrease in bath water temperature, as much as 8°C, does not compensate the short-term exposure to induce a decrement in human performance capacities. As a result of the findings, further investigations are needed to understand what the paramount component in neuromuscular function is related to cold temperature exposition.

## Data Availability

The raw data supporting the conclusion of this article will be made available by the authors, without undue reservation.
